# Skin treatment with non-thermal plasma modulates the immune system through miR-223-3p and its target genes

**DOI:** 10.1080/15476286.2024.2361571

**Published:** 2024-06-03

**Authors:** Annika Engel, Nicole Ludwig, Friederike Grandke, Viktoria Wagner, Fabian Kern, Tobias Fehlmann, Georges P. Schmartz, Ernesto Aparicio-Puerta, Dominic Henn, Barbara Walch-Rückheim, Matthias Hannig, Stefan Rupf, Eckart Meese, Matthias W. Laschke, Andreas Keller

**Affiliations:** a Chair for Clinical Bioinformatics, Saarland University, Saarbrücken, Germany; b Department of Human Genetics, Saarland University, Homburg/Saar, Germany; c Center for Human and Molecular Biology, Saarland University, Homburg/Saar, Germany; d Department of Clinical Bioinformatics (CLIB), Helmholtz Institute for Pharmaceutical Research Saarland (HIPS), Helmholtz Centre for Infection Research, Saarbrücken, Germany; e Department of Plastic Surgery, UT Southwestern Medical Center, Dallas, TX, USA; f Center of Human und Molecular Biology (ZHMB), Virology & Immunology, Saarland University, Homburg/Saar, Germany; g Clinic of Operative Dentistry, Periodontology and Preventive Dentistry, Saarland University, Homburg/Saar, Germany; h Institute for Clinical and Experimental Surgery, Saarland University, Homburg/Saar, Germany

**Keywords:** Non-thermal plasma, miRNA, single blood cell sequencing, wound healing, cell adhesion, miR-223-3p

## Abstract

Non-thermal plasma, a partially ionized gas, holds significant potential for clinical applications, including wound-healing support, oral therapies, and anti-tumour treatments. While its applications showed promising outcomes, the underlying molecular mechanisms remain incompletely understood. We thus apply non-thermal plasma to mouse auricular skin and conducted non-coding RNA sequencing, as well as single-cell blood sequencing. In a time-series analysis (five timepoints spanning 2 hours), we compare the expression of microRNAs in the plasma-treated left ears to the unexposed right ears of the same mice as well as to the ears of unexposed control mice. Our findings indicate specific effects in the treated ears for a set of five miRNAs: mmu-miR-144-5p, mmu-miR-144-3p, mmu-miR-142a-5p, mmu-miR-223-3p, and mmu-miR-451a. Interestingly, mmu-miR-223-3p also exhibits an increase over time in the right non-treated ear of the exposed mice, suggesting systemic effects. Notably, this miRNA, along with mmu-miR-142a-5p and mmu-miR-144-3p, regulates genes and pathways associated with wound healing and tissue regeneration (namely ErbB, FoxO, Hippo, and PI3K-Akt signalling). This co-regulation is particularly remarkable considering the significant seed dissimilarities among the miRNAs. Finally, single-cell sequencing of PBMCs reveals the downregulation of 12 from 15 target genes in B-cells, Cd4+ and Cd8+ T-cells. Collectively, our data provide evidence for a systemic effect of non-thermal plasma.

## Introduction

Non-thermal plasma (NTP), a partially ionized gas consisting of a mixture of ionized atoms, molecules, radicals, and photons, has been studied for clinical application in recent years. Technical applications include decontamination of sensitive instruments [1,2] due to its antibacterial effect [3–6]. Additionally, NTP treatment has been investigated in anti-tumourtherapy [7–9]. Interestingly, NTP-induced apoptosis was shown in head and neck cancer (HNC) cells via accumulation of reactive oxygen species *in vitro* and inhibited growth of HNC tumours *in vivo* [10,11]. However, one of the most explored applications of NTP treatment is wound healing. Recently, Wang *et al*. demonstrated inhibited scar formation after surgery [12]. Additionally, Isbary *et al*. reported a reduction of the bacterial load in chronic infected wounds [13]. Preliminary *in vitro* experiments showed that epithelial cells display an increased proliferation when exposed directly to NTP and also when exposed indirectly via NTP-treated culture medium [14–16], providing a potential mechanism underlying the improved wound healing processes. Considering stages of wound healing from inflammation to tissue formation and remodelling [17], NTP treatment supports wound recovery by stimulating the contribution of monocytes in the first stage [18]. The exact mode of action of NTP has not been fully identified and the knowledge on molecular processes underlying the improved wound healing and other positive effects of NTP are only partially understood. The aforementioned studies suggested the stimulation of the immune system as a systemic effect that might add to the local anti-microbial effects of NTP.

Beyond proteins, coding genes and metabolites, non-coding RNAs are well known to play an important role in processes related to the immune system. Among those, microRNAs (miRNAs) are a particular class of small non-coding RNAs, which are expressed in the course of a specific disease or in a cell-specific way [19–24]. The mechanisms how miRNAs are transcribed and processed remain an active field of research [19] but even basic principles remain still incompletely understood [25]. Large-scale databases such as miDIP suggest however over 46 million interactions between miRNAs and genes [26]. Databases such as miRTarBase collect over 2 million of verified miRNA gene interactions [27]. Such comprehensive resources are partially created using curated functional evidence. As an example, the role of infections and how pathogens can hijack miRNAs to suppress innate immunity of the host are known [28], indicating their importance in immune responses. Performing microRNA studies should typically also include an investigation of the target effects to improve a lacking understanding of mechanism in the light of the suggested wide variety of roles for miRNAs [29].

We thus hypothesize that NTP treatment has **(a)** a local influence on miRNA profiles that **(b)** potentially extends to a systemic influence and **(c)** thereby alters gene expression in mediators such as blood cells. To test this hypothesis, we study the effect of NTP treatment on mouse auricular skin. Because of the known effect of NTP treatment in wound healing, we measure differences in miRNA expression in treated versus untreated skin samples using deep sequencing. By including five different timepoints up to 120 minutes after the exposure, we are able to analyse immediate and time-dependent changes in miRNA profiles. Because of potential systemic regulatory effects on target genes, we finally carry out single-cell RNA sequencing on peripheral blood mononuclear cells (PBMC)s.

## Results

### Non-thermal plasma treatment has a limited global impact on the miRNA repertoire

**Figure 1.**
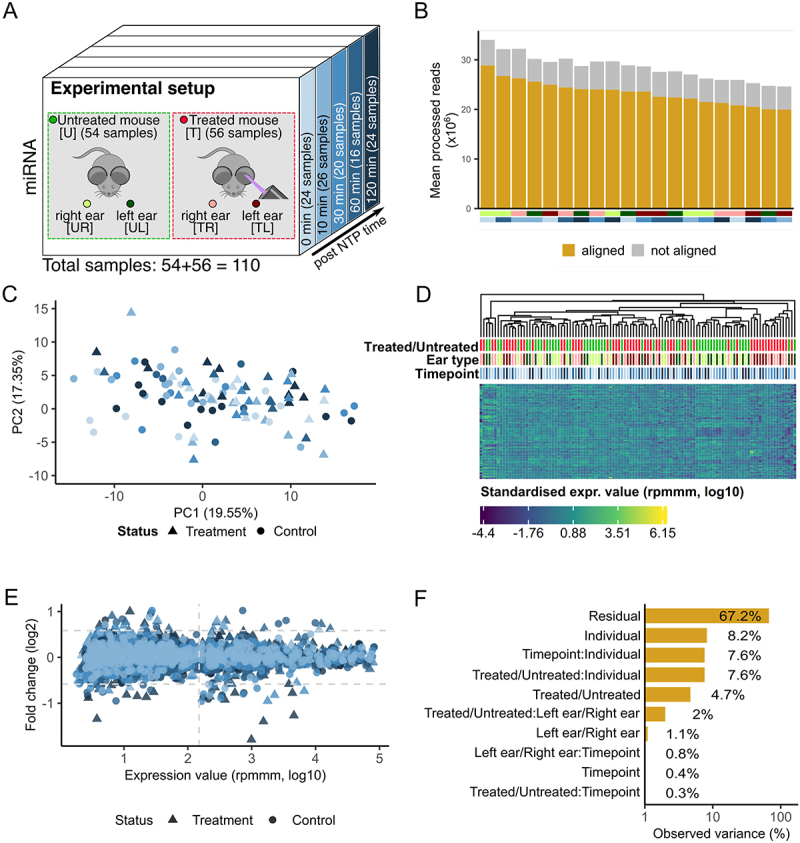
Study setup and expression variance analysis. (A) the data set consists of 55 mice, 28 of these were treated and 27 serve as a control group. Two samples were taken from each of the 55 mice – one from the skin of the left ear and one from the skin of the right ear. For the treated mice the left ear was exposed to NTP, and the samples were obtained at 5 different timepoints (0 minutes, 10 minutes, 30 minutes, 60 minutes, and 120 minutes). (B) the number of processed reads and the proportion aligned to the mouse genome. The bottom annotation indicates the sample type and timepoint. (C) Principal Component Analysis of the miRNA expressions. Outliers are excluded for visualization purpose. Colouration is done according to the five timepoints. Shapes indicate the treated and untreated mice. (D) Heatmap of the top 100 expressed miRNAs (log10) with hierarchical clustering. The top annotation rows provide information regarding the treatment status, the sample type and the timepoint. (E) Scatter plot of the FC (log2) of each timepoint to the minimal timepoint (0 minutes) versus the expression of each miRNA (log10). Coloured according to the five timepoints. The shapes correspond to treated and untreated mice. We differentiate between regulated and deregulated (FC ≥ 1.5 or FC ≤ 1/1.5) miRNAs according to the horizontal dashed lines. We assume that miRNA expressions left of the empirically determined threshold are dominated by noise. (F) Principal Variance Component Analysis of the miRNA expressions (log10). ‘Residual’ corresponds to the variance not covered by the properties.

To investigate the effects of non-thermal plasma (NTP), we select mouse ears as a suitable model system and analysed four distinct subsets: the left and irradiated ear of treated mice (TL), the right ear of treated mice (TR), the left ear of untreated mice (UL), and the right ear of untreated mice (UR). For clarity, we refer to these four groups as the sample types throughout the following sections. To capture the time-dependent processes, we consider miRNA bulk samples from five timepoints after the treatment (Figure 1A). A detailed description of the study setup is given in the method section.

As our initial experimental readout, we choose small RNA deep sequencing. After stringent quality control and adapter trimming, we include an average of 30 million reads per sample (Figure 1B). On average 25 million reads align with the mouse reference genome. Notably, no significant difference was observed within the four groups in terms of quality filtering, mapping to the reference genome, or mapping to mouse miRNAs (*Supplemental* Figure S1A). Altogether, we obtain 1966 miRNAs of which 495 remain as stably expressed in the data set.

Performing a Principal Component Analysis (PCA) with a global clustering of the miRNA expression data including all samples, we find no discernible clustering patterns based on treatment or timepoints (Figure 1C), arguing against a potential overarching molecular effect of NPT. To further explore the data and to identify less pronounced effects, we next conduct hierarchical clustering analysis for the top 100 miRNAs with the highest coefficient of variance (CV) across all samples (Figure 1D). Consistent with the PCA-based clustering results, we do not observe clear differentiation between treated and untreated samples, the different sample types, or the timepoints.

To check whether effects for single miRNAs exist at all, we calculate the fold changes (FCs) for all samples from treated and untreated mice separately across the timepoints (10 min vs. 0 min, 30 min vs. 0 min, 60 min vs. 0 min, 120 min vs. 0 min, Figure 1E). This analysis suggests a stronger effect in the treatment as compared to the control group, both, for miRNAs close to the background but also for highly abundant ones. Because of the large number of analyses (495 miRNAs, 4 timepoint comparisons for two groups) none of the markers in this global analysis remains significant following adjustment for multiple testing.

We thus ask for those factors yielding the overall largest impact on the expression levels using principal variance component analysis (PVCA). While PVCA is typically used to identify batch effects, we utilize it to uncover high-impact properties in our data set. We consider the attributes ‘Treated/Untreated’, ‘Left ear/Right ear’, ‘Timepoints’, and combinations of these attributes as potential factors affecting the expression levels. Additionally, we include the information that two samples always originate from the same mouse as a final attribute, ‘Individual’ (Figure 1F). The largest portion of the variance lies within the Residuals, indicating factors not explained by our experimental setup. We observe that the most substantial variance in the data set is associated with the ‘Individual’ property (8.2%), which is a common result pinpointing intra-individual variability. The combination of ‘Individual’ with ‘Treated/Untreated’, however, still significantly contributes to the overall data variance (7.6%). In a similar manner, the ‘Timepoint’ adds 7.6%. One notable result of the PVCA is that the left/right ear adds twice as much to the overall variance if considered in combination with the treatment.

In summary, our findings suggest that treatment with NTP has a limited influence on the global composition of miRNAs. However, it is important to note that this result does not imply the absence of changes in the abundance of individual miRNAs. In fact, the PVCA suggests significant differences between treated and untreated mice, especially if considered in the context of the timepoint and with respect to the left and right ear. This calls for a more focused analysis for each miRNA in the four sample types.

### Comparison between treated and untreated mice yields significant upregulated miRNAs

**Figure 2.**
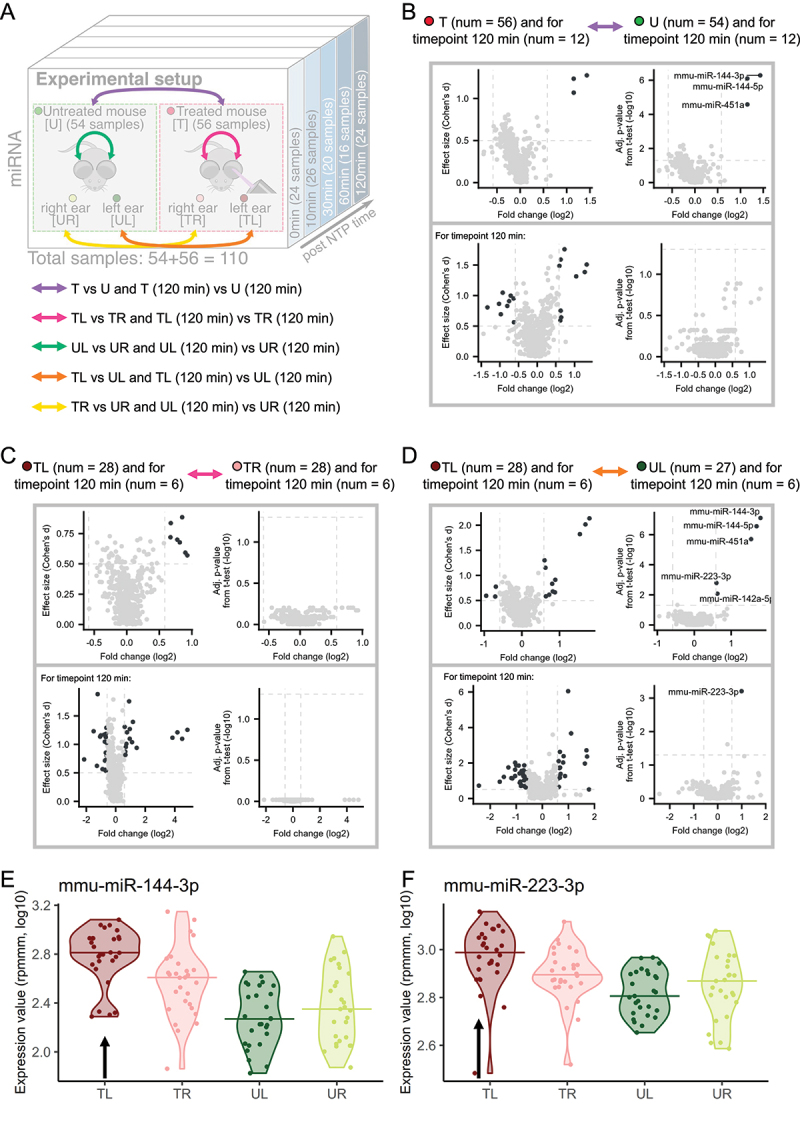
Group-based comparisons of miRNA expression. (A) the experimental setup with additional arrows indicating the comparisons we are investigating. (B) T versus U mice comparison. The effect size plots are located on the left side and Volcano plots on the right side of the different boxes with the comparisons from Figure 2a. The upper plots include all samples over all timepoints and the comparison for only the timepoint 120 minutes is depicted in the lower part of the box. Deregulated miRNAs with effect size ≥ 0.5 or p-value <0.05 (t-test, -log10) are highlighted in dark grey. (C) TL versus TR mice comparison analogous to Figure 2b. (D) TL versus UL mice comparison analogous to Figure 2b. (E) Violin plot for mmu-miR-144-3p. Each violin corresponds to a sample type and the horizontal lines indicate the medians. The black arrow marks the violin corresponding to the treated ear. (F) Violin plot for mmu-miR-223-3p analogous to Figure 2e.

For the four groups, we thus perform ear-wise comparisons within and between the treated and untreated cohorts and compare the results to each other (Figure 2A). Specifically, to identify miRNAs which are influenced by the treatment, we first compare all samples obtained from the treated to all samples from untreated mice since treatment accounts for the highest variance in the PVCA analysis after excluding all properties related to the individual. Of note, we compute not only the fold changes (FC) and the adjusted p-values (t-test) for the comparison but also the effect sizes (Cohen’s d). Our analysis reveals that three miRNAs mmu-miR-144-3p, mmu-miR-144-5p and mmu-miR-451a are more than 1.5-fold upregulated in treated mice and have a high effect size (≥0.5) and a significant adjusted p-value (Figure 2B; upper part). When considering only the last timepoint (120 minutes) we calculate the overall largest effect sizes for a higher number of miRNAs (Figure 2B; lower part). However, none of the miRNAs remain significant following adjustment. In the light of stronger effect sizes, the larger p-value may be explained by the smaller group sizes in this comparison (*n* = 24).

To make the comparison even more specific, we split the samples into the four sample types (TL, TR, UL, UR) and consider all combinations between treated and untreated with each side of the ear. Interpreting the results, it is important to keep in mind that NTP was only applied to the left ear. We start our analysis by comparing left and right ears first in the untreated (*Supplemental* Figure S2A) and then in the treated mice (Figure 2C). The results of the comparison of the left ear and the right ear within the untreated mice reveal no deregulated miRNAs, even if the latest timepoint is considered separately. In contrast, in the treated mice we identify upregulated miRNAs in the left ear compared to the right ear, even though they miss the significance level after adjustment for multiple testing. Focusing only on the 120 minutes timepoint confirms the result: we detect deregulated miRNAs, yet these do not prove significant following adjustment for multiple testing. We consequently investigate if the observed upregulation between treated and untreated samples can also be seen when comparing the left ears of treated versus the left ears of untreated mice (Figure 2D). In this comparison, we observe the same three miRNAs as above, i.e. mmu-miR-144-3p, mmu-miR-144-5p and mmu-miR-451a, to be of considerable effect size and significance level. In addition, two new candidates, mmu-miR-142a-5p and mmu-miR-223-3p, emerge for further investigation. When only looking at the 120 minutes timepoint, only mmu-miR-223-3p remains significantly upregulated.

Considering these results, we exemplarily compare the five normalized expression values for mmu-miR-144-3p and mmu-miR-223-3p across the four sample types directly to each other (Figures 2E,2F). The medians of the samples of the treated mice show a higher expression compared to the untreated ones. Since only the left ears were stimulated with NTP and we find no significant miRNAs between the right ears of the treated and right ears of the untreated mice (*Supplemental* Figure S2B), we suggest that the observed upregulation is caused by the application of NTP. Overall, this argues for the hypothesis of a systemic effect of the treatment.

To gain a better understanding of the underlying effects in the data set, we thus take a closer look at the dynamic variability dependent on the time after exposure to the NTP as a potential key factor.

### Detailed view on significantly upregulated miRNAs

**Figure 3.**
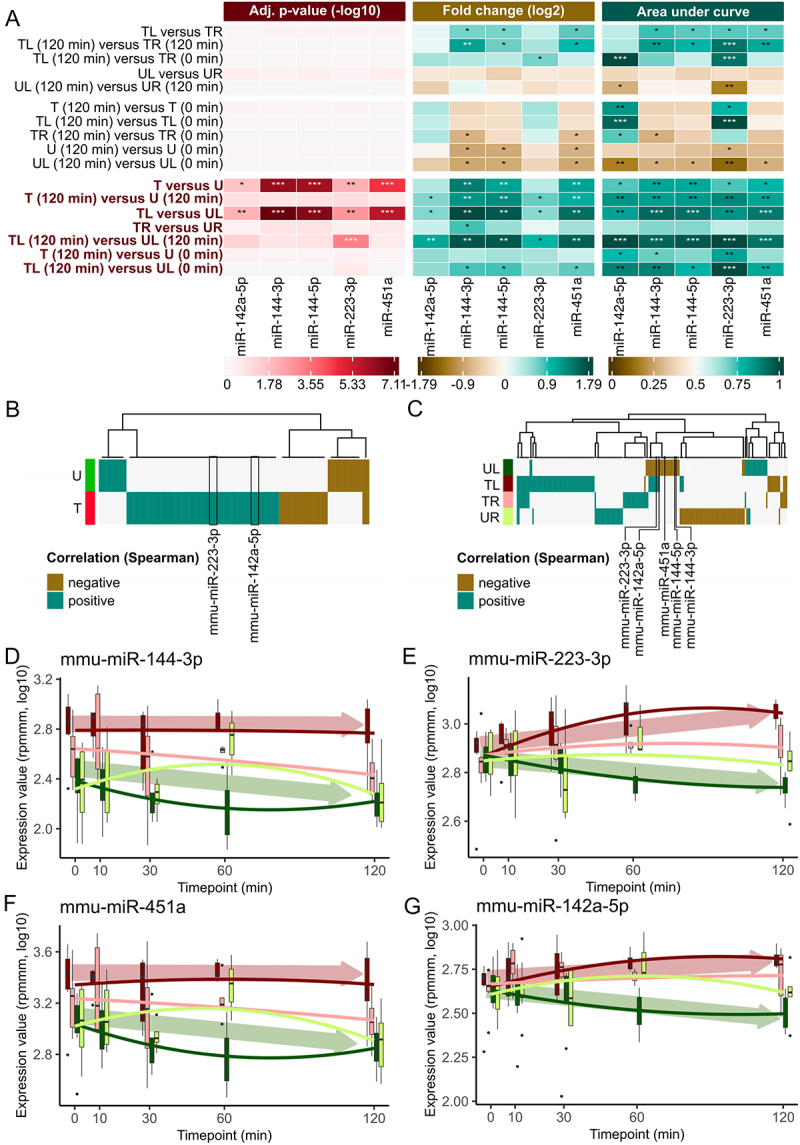
Group-based comparisons and time evolution of miRNA expression for mmu-miR-142a-5p, mmu-miR-144-3p, mmu-miR-144-5p, mmu-miR-223-3p and mmu-miR-451a. (A) Heatmaps of adjusted p-value from t-test (−log10), FC (log2) and AUC for manually selected comparisons. A p-value of under 0.05 is indicated by a *, of under 0.01 by ** and under 0.001 by ***. A deregulation of miRNAs by a factor of 1.5 is marked with a * and a factor of 2 with **. An AUC value between 0.7 and 0.8 or between 0.3 and 0.2 is marked by *, between 0.8 and 0.9 or between 0.2 and 0.1 is marked by ** and higher than 0.9 or lower than 0.1 is marked by ***. Promising comparisons between the treated and untreated mice are highlighted in red on the left side. (B) Heatmap plot of Spearman correlation between miRNA expressions and timepoints for the samples of the treated and untreated mice. Only miRNAs with a correlation lower than − 0.3 or higher than 0.3 are depicted. Correlation values are highlighted as negative correlated if the correlation values are < −0.3 and positive correlated if the correlation values are > 0.3. (C) Heatmap plot of Spearman correlation between miRNA expressions and timepoints for the samples of the four sample types analogous to Figure 3b. (D) Box plot over all five timepoints for mmu-miR-144-3p for each of the four sample types. The ends of the box denote the 25^th^ percentile and the 75^th^ percentile and the median is given as a horizontal line. The solid lines show a polynom of second degree fitted to the expression values using least-squares. Arrows indicate the overall trend of samples from treated (red) and untreated (green) mice. (E) Box plot for mmu-miR-223-3p analogous to Figure 3d. (F) Box plot for mmu-miR-451a analogous to Figure 3d. (G) Box plot for mmu-miR-142a-5p analogous to Figure 3d.

In the subsequent analysis, we focus our investigation on the five miRNAs that exhibit significant deregulation. When comparing samples within the same sample type but at the timepoints furthest apart from each other (Figure 3A, upper part), we observe a downregulation of miRNA expression in all the samples from the untreated mice and just the samples from the left ear from the untreated mice when considering the timepoint difference between 120 and 0 minutes. This downregulation suggests a general change in miRNA expression over time, potentially influenced by the anesthesia. Interestingly, a similar downregulation is also detected in the samples taken from the right ears of the NTP-treated mice. Furthermore, no significant deregulation is observed between the left and right ears, indicating a consistent behaviour of both ears. However, the downregulation over time does not reach statistical significance, suggesting a limited impact of the anaesthesia. In contrast, we observe a significant upregulation of all five miRNAs in the treated samples compared to the untreated samples (Figure 3A, lower part). This change is consistent across all samples from the treated mice compared to all samples from the untreated mice. Moreover, this upregulation is also evident when comparing the left ears of the treated mice to the left ears of the untreated mice. However, we detect no significant difference in miRNA expression when comparing the right ears of the treated mice to the right ears of the untreated mice.

We thus calculate the Spearman correlation between the miRNA expression and the time following exposure to NTP (Figures 3B,3C). For both, treated and untreated samples, we observe mainly positively correlated miRNAs. In the sample-type-specific investigation, however, we find that the majority of miRNAs in the treated mice are positively correlated with the progression of time, while in the untreated mice they are negatively correlated. Notably, the five significant miRNAs from the previous analysis are all negatively correlated with time in the samples from untreated mice, even though not below our empirically determined threshold (−0.3). MiRNAs mmu-miR-223-3p and mmu-miR-142-5p present a positive correlation with time in the samples from treated mice (corr = 0.36 and corr = 0.35) and in the samples from left ears of treated mice (corr = 0.61 and corr = 0.49). Such a systemic effect, however, should become most obvious in the detailed time analysis and become more pronounced over time (Figures 3D-3G). We confirm the general trend of microRNAs being higher abundant in treated samples than untreated ones in the majority of timepoints. Resolving the expression over all timepoints does not indicate a distinct behaviour for mmu-miR-144-3p. In contrast, mmu-mir-223-3p, which is the only miRNA significantly upregulated for timepoint 120 minutes in the comparison between TL and UL, presents a monotonous increase from 0 minutes until 30 minutes and remains stable, highlighting it as a most likely systemic miRNA. Altogether, this narrow set of five miRNAs with potential effects opens the question on regulatory mechanisms affecting the expression of genes and pathways.

### Upregulated miRNAs regulate wound healing and are enriched in immune and blood cells

**Figure 4.**
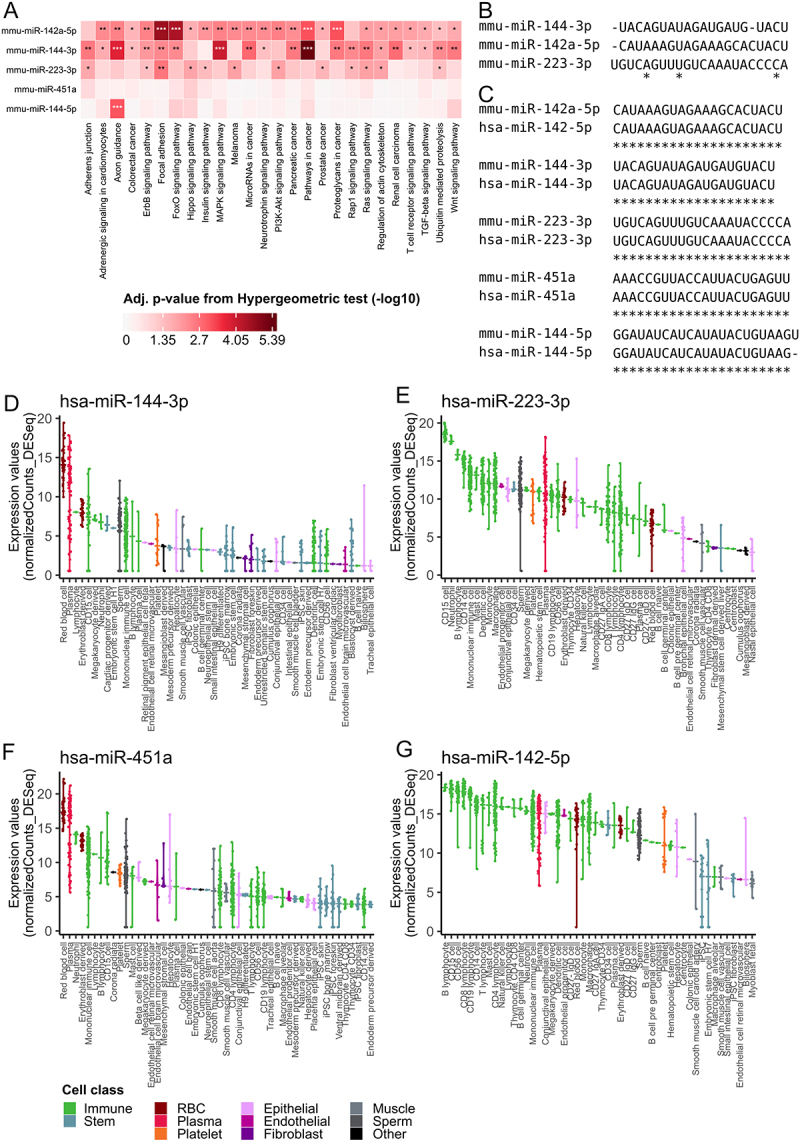
Connected signalling pathways and miRNA occurrence in human cells. (A) Heatmap of adjusted p-value from Hypergeometric test (−log10) for pathways originating from the KEGG database related to the five upregulated miRNAs [30]. We use predicted union and a pathway is shown if it has at least two significant miRNAs. One or more asterisks indicate the significance (cf. Figure 3a). (B) a multiple sequence alignment for the miRNAs mmu-miR-144-3p, mmu-miR-142a-5p and mmu-miR-223-3p. Asterisks highlight the region for which the miRNAs exhibit the same base. (C) an alignment of the human and mouse related miRNA for the five significantly upregulated miRNAs. Asterisks highlight the region for which the miRNAs exhibit the same base. (D) Violin plot for hsa-miR-144-3p from a previously published data set [31]. Every violin depicts normalized (DESeq2) expression data for specific cell types. Cell types are sorted descending by their medians, are cut after the first 50 and grouped into cell classes highlighted by different colours. Higher values indicate a more frequent occurrence of the miRNA in the respective cell type. (E) Violin plot for hsa-miR-223-3p [31]. (F) Violin plot for hsa-miR-451a [31]. (G) Violin plot for hsa-miR-142-5p [31].

To connect our miRNA findings to causal mechanisms, we perform a pathway analysis using the miRPathDB [30] resource (Figure 4A). Especially mmu-miR-142a-5p, mmu-miR-144-3p and mmu-miR-223-3p indicate a similar set of pathways connected to wound healing and tissue regenerationfor example, the ErbB, FoxO, Hippo and PI3k-Akt signalling pathways [32–34]. The MAPK signalling pathway, which shows to be especially relevant in association with mmu-miR-144-3p, is known to be activated by NTP treatment [18]. In addition, the Adherens junction, Rap1 and Ras pathways exhibit effects regarding the cell adhesion and affiliations to epithelial tissue [35,36]. Furthermore, Ras and TGF-beta pathways are involved in cell-cycle control and apoptosis [37,38]. A co-regulation of miRNAs to similar sets of genes and thus of similar pathways is not surprising and well-known, especially if the seeds of the miRNAs are similar. But there are also examples of miRNAs with very different seeds regulating similar pathways [39]. We thus compute a multiple sequence alignment for the three miRNAs sharing similar target pathways (Figure 4B). While miR-144-3p and miR-142a-5p indeed share some similarity in the relevant mature miRNA sequences, miR-223-3p has no seed similarity to the former. This again provides further evidence for a biologically relevant function. At the same time, it immediately poses the question how these miRNAs, and especially miR-223-3p, mediate a potential systemic effect.

To further improve our understanding of the causal mechanisms, we investigate the expression of the miRNAs across different cell types. Because of the homology of mouse miRNAs to humans and a significantly better data basis for human miRNAs in cell types, we perform this analysis on human cell lines. For four of the significantly upregulated miRNAs (miR-142(a)-5p, miR-144-3p, miR-223-3p and miR-451a) the computed alignments of their mouse and human sequences map in every position (Figure 4C). Accordingly, we are able to use a very comprehensive data set of miRNAs across human cell types [31] as a reference resource. We individually examine the expression of hsa-miR-144-3p, hsa-miR-223-3p, hsa-miR-451a and hsa-miR-142-5p at the cell type level (Figure 4D-G). We observe that these four miRNAs are common in blood (red coloured classes, e.g. red blood cells, plasma, and erythroblast-derived cells), immune (green coloured, e.g. CD15+ cells, neutrophil and B lymphocyte and CD56+ cells) and skin cells (pink coloured classes, e.g. hepatocyte, endothelial progenitor cells, conjunctival epithelial cells, Beta cell like derived cells and Endothelial cell retinal microvascular cells).

The significant abundance in skin cells is expected as this is the main specimen of the samples. The dysregulation of miRNAs mainly expressed in immune cells circulating in blood argues for a systemic effect. The principal occurrence of hsa-miR-144-3p and hsa-miR-451a in blood cells and the expression difference between the four experimental groups already visible at the earliest timepoint (0 minutes) (Figures 3D,3F) allows for the hypothesis that the miRNAs may show a direct response to the NTP treatment. In contrast, no difference for mmu-miR-223-3p and mmu-miR-142a-5p at 0 minutes exists (Figures 3E,3G) and the deregulation occurred delayed. This hints towards a dysregulation within the immune system occuring after an initial effect mediated by afore mentioned cell types within the treated tissue. To check our hypothesis of a systematic response to NTP treatment mainly within blood and especially circulating immune cells in blood, we take a closer look at our miRNA candidates and their occurrence in blood cells. For this purpose, we first take into account the data set presented by Juzenas *et al* [40]. This data set offers a comprehensive, cell-specific miRNA catalogue of selected peripheral blood mononuclear cell (PBMC) types (including CD4+ cells, CD8+ cells, B cells (CD19+ cells) and NK cells (CD56+ cells)). Since the data set again refers to human miRNAs, we consider the same four miRNAs as above with complete sequence homology between human and mouse. We observe that hsa-miR-223-3p, hsa-miR-451 and especially hsa-miR-142-5p are strongly expressed in the cell types shown (NK cells, B cells, CD4+ cells and CD8+ cells) (*Supplemental* Figure S3A). Therefore, we decide to perform a further analysis to investigate the changes in PBMCs under NTP treatment. Here, single-cell RNA sequencing provides the best option to evaluate the changes over time for the different immune cell types.

### Single immune cell sequencing supports downregulation of targeted genes via miRNA overexpression

**Figure 5.**
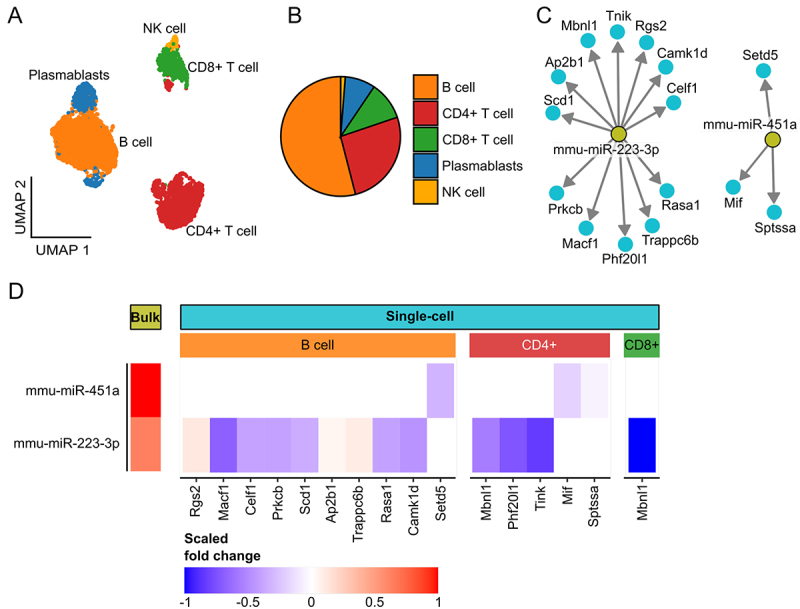
Single-cell quality assessment and associated gene miRNA expression changes. (A) UMAP of single-cell PBMC data coloured by cell type. (B) Cell type proportions in the data set (total: 5794 cells). (C) a graph network to display some of the target genes for miRNAs mmu-miR-223-3p and mmu-miR-451a provided by mirTarbase [27]. The target genes are selected by intersecting all target genes with the significant genes (p-value <0.05) from the single-cell data set. (D) Heatmap plot shows scaled FCs (scaled by the maximal absolute FC in the bulk and single-cell data, respectively) for the miRNAs (bulk data set) and some of their target genes (single-cell data set) from Figure 5c. Red yields an up- and blue a downregulation.

We thus perform single-cell sequencing of peripheral blood mononuclear cells (PBMCs) using the same experimental setup. Because the largest impact in our time-series experiment is present at the latest time point and potential regulatory mechanisms might take additional time, we perform the analyses for the latest time point in the study. For this experiment, we use a second cohort of mice and compare blood from two treated to two untreated mice. We sequence 7503 peripheral blood mononuclear cells (PBMCs) and after quality control end up with 5794 high-quality cells. The cell-type annotation highlights five different main cell types: B cells, CD4+ T cells, CD8+ T cells, plasmablasts and NK cells (Figures 5A,5B). Due to the fact that the single-cell method we use to sequence the PBMC data does not allow for quantifying mature miRNAs, we identify the target genes of the four miRNAs with the tool MirTarBase [27] to verify the previously seen effects. In a subsequent differential expression analysis between cells from treated and untreated mice, we find a downregulation for 12 of the 15 target genes linked to the deregulated miRNAs mmu-miR-223-3p and mmu-miR-451a (Figures 5C,5D). Most of the target genes show a deregulation in B cells (7 down and 3 upregulated), followed by CD4+ T cells (5 downregulated) and CD8+ T cells (1 downregulated). Mbnl1 that is targeted by miR-223-3p shows a downregulation both in CD4+ and CD8+ T cells and has been previously found to be involved in the differentiation of fibroblasts during wound healing [41]. This supports the assumption that a treatment with NTP influences both the level of the miRNA and their respective target genes.

## Discussion & Conclusion

In this study, we systematically explored the effect of NTP treatment on mouse tissue samples. While the exact mode of NTP action has not been fully identified, previous studies showed that epithelial cells display an increased proliferation after NTP exposure and reported an inhibited scar formation in *in-vitro* experiments [12,15,16]. To examine the underlying molecular processes contributing to improved wound healing following NTP treatment, we analysed non-coding RNA from irradiated auricular skin and performed single-cell sequencing of blood.

Unsupervised hierarchical clustering and PCA as a first attempt to group the samples according to their known properties, e.g. treated/untreated, left/right ear or the timepoint, did not yield the anticipated result. Yet, a subsequent PVCA indicated a local influence by yielding promising comparisons between sample subsets, e.g. treated versus untreated left ears. From these comparisons, we obtained five significantly upregulated miRNAs (mmu-miR-144-5p, mmu-miR-144-3p, mmu-miR-142a-5p, mmu-miR-223-3p and mmu-miR-451a).

To reveal a potential systemic influence of the treatment, we investigated this set of miRNAs in a two-fold manner. First, we presented a relation between the upregulated miRNAs and pathways directly linked to wound healing and associated processes like tissue regeneration and cell cycle control. Additionally, mmu-miR-144-3p exhibited a connection to the MAPK signalling pathway which was reported to be induced by NTP treatment in human monocytes [18].

These findings fit to the second part of the investigation where we showed a correlation of miR-223-3p to the NTP treatment. This effect seemed not to be limited to the treated site but systemic. Several recent studies connected the deregulation of miR-223-3p to healing processes. Cheng *et al*. reported that mmu-miR-223-3p increases the muscle regeneration in the early stage after an injury in mice [42]. They attributed the increase on the regulation of inflammation via the suppression of the target gene interleukin-6 (Il6). Effects concerning the inflammation regulation were also shown to suppress necroptosis in ischaemic hearts [43]. A study investigating the healing of human gingiva reported hsa-miR-223-3p as most upregulated miRNA [44]. In addition, Wu *et al*. presented an increase in hsa-miR-223-3p over the timespan of 21 days in patients with two kinds of fractures and accounted the effect to the regulation of cell viability and cell apoptosis [45].

Additionally, we were able to roughly sketch out the timing of the mmu-miR-223-3p upregulation induced by NTP, which reached the highest level after 30 minutes after treatment. Altogether, this suggests a connection to the inflammatory phase of wound healing occurring in the first few hours after an injury.

To support our findings, we prepared a second data set with single-cell PBMC data to collect more information based on immune cells in the blood. Especially for some target genes of mmu-miR-223-3p (provided by miTarBase [27]) we observed a downregulation in the differential expression analysis. This opposite behaviour compared to the upregulation of the miRNA supports the assumption of an effect of NTP treatment on a RNA-level. We detected a downregulation of the gene Mbnl1 that is targeted by miR-223-3p in the blood. Although previous publications linked an increased expression of Mbnl1 in skin [41] and heart tissue [46] to an improved wound healing, this does not directly contradict a possible improvement of the wound healing due the downregulation of Mbnl1 in the blood after NTP treatment. Two factors that greatly influence the observed changes in gene expression are the used cells, as changes in the gene expression in blood often differ from the gene expression in other tissues, and the fact that previous publications were focused mainly on the effect in damaged tissue. Instead, we focused on the effect of NTP in healthy, non-injured mice.

Summarizing the bulk and the single-cell study, on the one hand, we found a set of upregulated miRNAs in a bulk experiment and a targeted gene in the subsequent single-cell analysis with reported effects in conjunction to wound healing processes. On the other hand, our analysis resulted in the connection to a signalling pathway, which was already reported in the context of NTP treatment. We conclude that NTP treatment induced some changes on miRNA-level, which might partially contribute to the supporting effects of NTP in wound healing, but further experiments are needed to confirm these conclusions and to decipher the exact mode of action.

## Limitations and further work

In the bulk study, we were not able to observe a local difference between left and right ears nor a significant deregulation over time. This could be attributed to the low sample density, with only four to seven samples per timepoint and sample type. Additionally, many effects of wound healing occur immediately after the injury, yet further stages occur in the span of several days[17]. For example, mmu-miR-223-3p was only significantly upregulated for the last timepoint (120 minutes). This suggests that a further study would benefit from more samples per timepoint and a longer study period. However, we chose our experimental parameters optimizing the balance between sufficient samples per time point and minimizing the number of animals sacrificed for the study. The latest time point of 120 minutes was chosen to prevent possible narcosis damage of the mice caused by an extended sedation.

For the validation of the bulk results, we only focused on single-cell sequencing of blood. Due to the constraints of available blood volume from the experimental subjects, we omitted complementary measurements of microvesicles. Nonetheless, our previous studies supported the hypothesis that microversicles are likely primary mediators of miRNA transport and signal transmission [47,48,50]. Furthermore, for a definitive exploration of the mechanisms contributed to a miRNA, e.g., mmu-miR-223-3p, an experimental setup could be considered where the development of the miRNA in question is artificially inhibited or prevented . Yet, such a tissue-specific knock-out remained out of the scope for this preliminary investigation. An additional experimental verification of the observed dysregulated genes, e.g. a qPCR analysis, would present a beneficial investigation but was left out considering the quality of our samples leveraging 10X Chromium technology. Although we showed that the miRNAs of interest are present in PBMCs, our single-cell data only represents gene changes, as standardised high-throughput methods for the quantification of mature miRNAs are not yet established but are part of ongoing research [49,50]

## Methods

### Bulk study

#### Study setup

We carefully selected a suitable model system that would allow for controlled experiments in a well-defined environment. The mouse ear emerged as an ideal choice due to its physiological characteristics, including the absence of hair, accessibility, and the presence of a well-established vasculature. By focusing on uninjured auricular skin as our model system, we aimed to study the direct effects of NTP without inducing unnecessary stress on the mice. This particular choice of model system offered several advantages. First, it enabled us to analyse the molecular processes occurring in healthy skin that were stimulated by exposure to NTP. Second, the lack of injury eliminated confounding factors associated with wound healing processes, allowing us to isolate the specific effects of NTP on healthy skin. Additionally, the ease of sampling from the auricular skin simplified the experimental procedures. To capture the dynamic changes induced by NTP exposure, we obtained samples from the treated mice immediately after the end of NTP treatment (0 minutes), as well as at 10, 30, 60, and 120 minutes post-treatment. Besides the samples from the treated mice (irradiated and un-irradiated ears) we considered a equally sized control group.

## Samples

All animal experiments were approved by the local authorities (Landesamt für Verbraucherschutz, Saarbrücken, Germany; permission number: 21/2018) and conducted in accordance with the European legislation on the protection of animals (Directive 2010/63/EU) and the National Institutes of Health (NIH) guidelines on the care and use of laboratory animals (NIH publication #85–23 Rev. 1985). The C57BL/6 wild-type mice used in this study were housed in groups on wood chips as bedding under a 12 h day/night cycle in the animal facility of the Institute for Clinical and Experimental Surgery (Saarland University, Homburg/Saar, Germany) with free access to water and standard pellet food (Altromin, Lage, Germany). To exclude age and gender as potentially confounding factors, we selected 3-month-old female mice (3.2 ± 0.3 months) for our study, which were assigned to either treatment or control group. All mice were anaesthetized by an intraperitoneal injection of ketamine (75 mg/kg body weight; Ursotamin®; Serumwerke Bernburg, Bernburg, Germany) and xylazine (15 mg/kg body weight; Rompun®; Bayer AG, Leverkusen, Germany). The left ear of the mice in the treatment group was irradiated with NTP for 10 minutes using an atmospheric pressure argon plasma jet kINPen Med [51] (Neoplas, Greifswald, Germany) with argon (Air Liquide, Düsseldorf, Germany) using a flow rate of 5 standard litres per minute (sL/m). The distance between the tip of the plasma effluent and the mucosa was between 5 and 10 mm. The plasma jet was applied in meander-like motion to prevent heating of the treated tissue. The mice of the control group were equally anaesthetized but remained unexposed to NTP. Right and left ears of treatment and control mice were collected after 0, 10, 30, 60, and 120 minutes, immersed in RNAlater for 12 hours at 4°C and subsequently frozen at -80°C. RNA was isolated using miRNeasy Mini kit (Qiagen, Hilden, Germany) after manufacturer’s recommendations. RNA quantity and quality was determined using Nanodrop (ThermoFisher Scientific, Waltham, MA, USA) and Bioanalyzer RNA Nano Chip Kit (Agilent Technologies, Santa Clara, CA, USA). Mean RNA Integrity Number (RIN) of the samples was 8.2 ± 0.9.

## Library preparation and sequencing

As our initial experimental readout, we chose small RNA deep sequencing which allows for high-resolution annotation of miRNAs. Small RNA sequencing libraries were prepared using MGIEasy Small RNA Library Prep Kit on the high-throughput MGI SP-960 sample prep system (MGI Tech, Shenzhen, China). According to the manufacturer’s protocols, the 3’- and 5’-adapters were ligated to the RNA. Afterwards, an RT primer including sample-specific barcodes was utilized to perform reverse transcription (RT). Amplification of the cDNA was achieved using a polymerase chain reaction (PCR) with 21 cycles. PCR products were subsequently size selected via AMPure Beads XP (Beckman Coulter, Brea, CA, USA). Size and concentration of the purified PCR products were determined using an Agilent DNA 1000 Kit (Agilent Technologies, Santa Clara, CA, USA) and Qubit™ 1X dsDNA High Sensitivity (HS) (ThermoFisher Scientific, Waltham, MA, USA), respectively. Fifteen samples and one performance control sample were pooled in an equimolar fashion in one sequencing library, which was circularized using the MGI Easy circularization kit. In total, we prepared 8 libraries composed of a total of 120 samples which were sequenced on DNBSEQ-G400RS High-throughput Sequencing Set (Small RNA FCL SE50) by BGI in Wuhan, China.

## Data analysis

The pipeline ‘miRMaster 2.0’ in standard settings [52] was used to process the sequencing data. Furthermore, only miRNAs were considered for further analysis and called expressed that exhibited more or equal than 5 raw reads in 100% of the samples of at least one sample type. We normalized the expression data to make the samples comparable. Therefore, we used the rpm-normalization integrated in the above-mentioned pipeline. This normalization considers the number of reads mapped to a miRNA and the total number of reads mapped to all miRNAs in units of million reads. In this work, we call this normalization rpmmm-normalization. For the further analysis, we used the filtered and normalized expression data, which consisted of 495 miRNAs. A total of 6 samples distributed over all sample types showed a vastly different behaviour in a preliminary analysis. We identified these samples as outliers and removed both samples of the corresponding mice for further analysis. In total, we obtained 110 samples after outlier removal.

Data analysis was performed using R in version 4.2.2 with the following packages: data.table in version 1.14.6, tidyverse in version 1.3.2, effsize in version 0.8.1, pROC in version 1.18, cowplot in version 1.1.1, reshape2 in version 1.4.4, ComplexHeatmap [53] in version 2.14.0, viridisLite in version 0.4.1, lme4 in version 1.1, readxl in version 1.4.1 RhpcBLASctl in version 0.21.

We always applied an unpaired two-tailed t-test. For the adjustment of p-values we used the Benjamini–Hochberg procedure. We used effect sizes to identify and specify quantifiable statements of an underlying effect between to subsets of a data set. To measure the effect size, which is done for every feature individually, we considered several measures such as fold change (FC), Cohen’s d and the area under the receiver operator characteristics curve (AUC). The FC is derived by building a ratio between two expression values. If the ratio is 1, this means that the two values are equal. If the ratio is <1 (>1), we know that the part we divided by, exhibits the higher (lower) value. We say that the change between the two values is down (up) directed. We called a miRNA deregulated if it passed in one direction a specified fold change threshold and significant deregulated if its adjusted p-value is lower than 0.05. We defined a miRNA as upregulated if FC ≥ 1.5 and downregulated if FC ≤ 1/1.5. Whereas the fold change just represents the ratio between two values, Cohen’s d uses a sophisticated ratio including the mean and the estimated variances of the two subsets calculated with the effsize package. We interpreted a feature as impactful if its Cohen’s d value ≥0.5. The third effect size we used was the AUC value. We took the expression values of the samples included in the comparison as a prediction and a binary value corresponding to the respective subset the sample belonged to as response. This allowed us to build an ROC curve. In this way, upregulated miRNAs achieved an AUC value close to 1 and downregulated ones close to 0. To determine the AUC value, we used the pROC package. All correlations were calculated using the Spearman rank correlation. For correlation analysis, only miRNAs exceeding the interval between −0.3 and 0.3 were marked as negatively or positively correlated. Principal Variance Component Analysis (PVCA) is a combination of principal component analysis (PCA) and variance component analysis (VCA). As a result, we obtained the proportion of variance for the available properties. The residual bar represented the remaining variance which is not covered by any of the given properties. The listed properties and their combinations presented their impact on the total variance in the data set. For clustering, we used hierarchal clustering with Euclidean distance and complete linkage. For the clustering of the expression data, we first standardized, which means the data were transformed to a mean of 0 with standard deviation of 1. To create the heatmaps, we used the ComplexHeatmap package. The data for the heatmap depicting pathways, originated from the tool miRPathDB [30], which outputs all significant KEGG pathways for the entered miRNAs. The values in the heatmap indicated the p-value in -log10-transform originating from the Hypergeometric test for the corresponding enrichment result.

## Single-cell transcriptome analysis

### Samples, library preparation and sequencing

The experimental setup for data collection was the same as for the bulk samples. As for the bulk samples, C57BL/6 mice from the Institute of Clinical and Experimental Surgery (Saarland University, Homburg/Saar, Germany) were used. All mice were anaesthetized as described above and, in the case of the treated mice, irradiated with NTP for 10 minutes. The plasma and the treatment itself were handled in the same way as for the bulk samples. Following a period of 120 minutes after irradiation for the treated mice and after the onset of anaesthesia for the untreated mice, blood (usually between 500 and 800 µL) was collected from the vena cava.

PBMC were isolated from heparin blood of four mice using standard Ficoll gradient. Cells were counted in a haemocytometer using Trypan blue for live/dead staining. Viability was >95% for all samples. Single-cell transcriptome analysis was performed using Chromium Next GEM Single Cell 3’ Kit v3.1 (10×Genomics) with 5,000 cells input after the manufacturer’s protocol. Generated single-cell libraries were sequenced by Novogene on an Illumina NovaSeq using PE150 sequencing.

## Data analysis

Reads were aligned against the mm10 genome (refdata-gex-mm10–2020-A) using the 10× Genomics CellRanger software (v.7.1.0) using a cut-off value of 200 UMIs.

For quality-control, SoupX [54] (v.1.6.1) was used to remove ambient cell-free mRNA contamination, cells with more than 10% mitochondrial reads, <200 features or > 2,500 features were removed using Seurat [55] (v.4.3.0). As a third quality-control step, doublets were removed using DoubletFinder [56] (v.2.0.3) using automatically determined (using the recommended settings) parameters nExp and pK.

The samples were aligned using Seurat integration with default settings and dimensionality-reduction was performed using PCA and UMAP on the first 20 dimensions. The data was clustered using the FindNeighbors and FindClusters Functions of Seurat at 0.8 resolution.

For the cell type annotation, a differential expression analysis using Seurats FindAllMarkers function (Wilcox) between each cluster and all other clusters was performed to identify marker-genes and the cells were annotated using known marker-genes. Clusters with the same cell type were merged.

Genes that were differentially expressed between treated and untreated mice were determined using the FindMarkers Function of Seurat with MAST [57]. Genes were selected if they had an adjusted p-value (Bonferroni correction) below 0.05.

## Supplementary Material

Supplemental Material

## Data Availability

All sequencing data are available for download from NCBI’s Gene Expression Omnibus [58] via GEO Series accession number GSE236802 (https://www.ncbi.nlm.nih.gov/geo/query/acc.cgi?acc=GSE236802)

## Code availability

The code that supported the analysis within this paper is available at https://github.com/CCB-SB/2023_mouse_ear_non-thermal_plasma

## Extended Data

Supplemental Figure S1: **(A)** The percentage of reads, which are mapped to miRNAs. The bottom annotation indicates the sample type and timepoint.

Supplemental Figure S2**: (A)** TR versus UR mice comparison. The effect size plots are located on the left side and Volcano plots on the right side of the different boxes with the comparisons from Figure 2A. The upper plots include all samples over all timepoints and the comparison for only the timepoint 120 minutes is depicted in the lower part of the box. Deregulated miRNAs with effect size ≥0.5 or p-value <0.05 (t-test, -log10) are highlighted in dark grey. (B) UL versus UR mice comparison analogous to Supplementary Figure S2a.

Supplemental Figure S3**: (A)** Adjusted (layout changes and display only four cell types) plot from the web tool of Juzenas et al. [40]. This data set is offering a comprehensive, cell-specific miRNA catalogue of selected PBMC types.
