# Supplementary material for: Skin treatment with non-thermal plasma modulates the immune system through miR-223-3p and its target genes
Source: RNA Biol. 2024 Jun 3;21(1):31–44. doi: 10.1080/15476286.2024.2361571 (PMC11152102; doi:10.1080/15476286.2024.2361571)
Supplement: Supplemental Material [file KRNB_A_2361571_SM2072.zip › supp_figures.pdf]

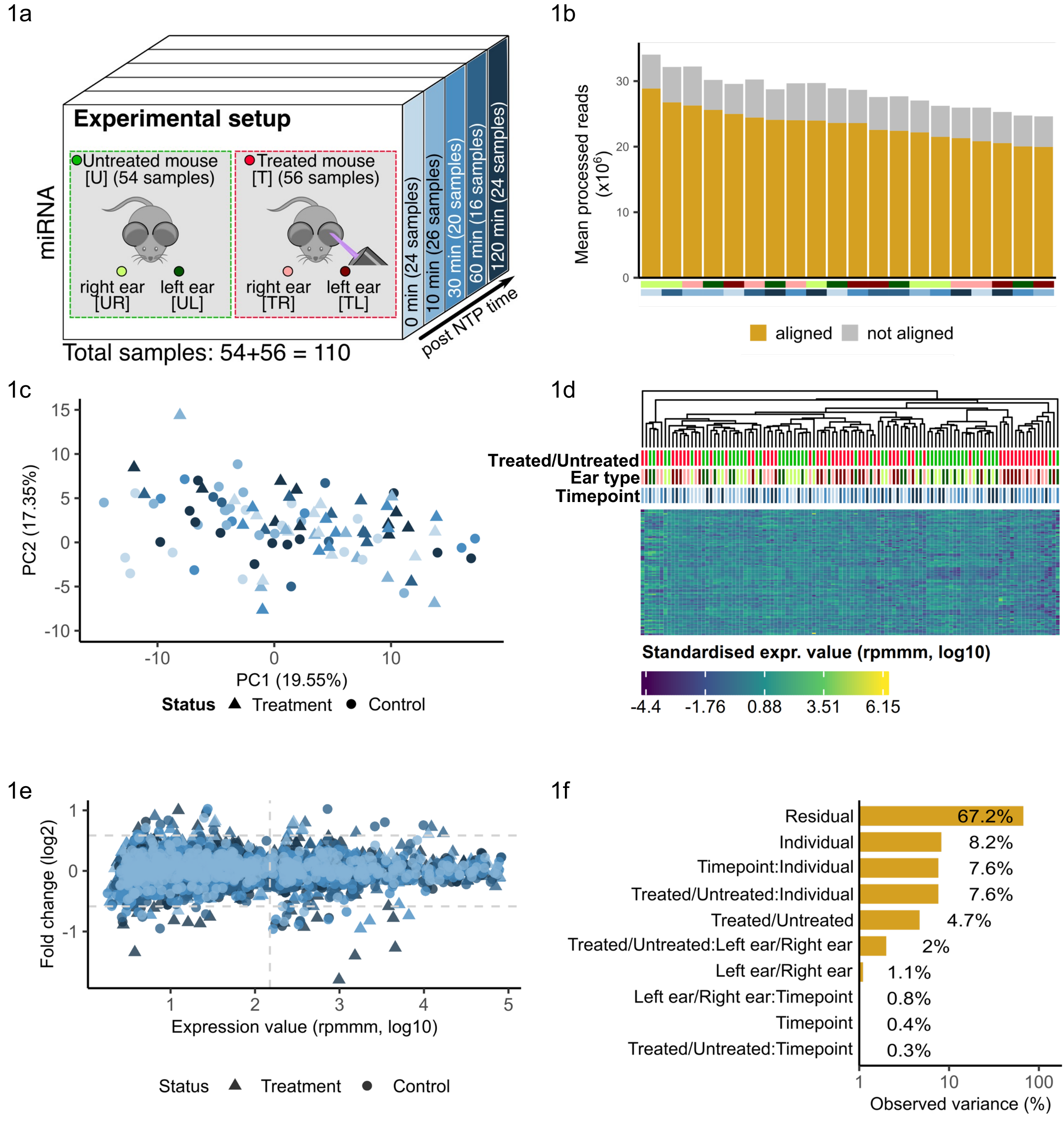
1a
Experimental setup
miRNA
Untreated mouse
[U] (54 samples)
right ear
[UR]
left ear
[UL]
Treated mouse
[T] (56 samples)
right ear
[TR]
left ear
[TL]
0 min (24 samples)
10 min (26 samples)
30 min (20 samples)
60 min (16 samples)
120 min (24 samples)
post NTP time
Total samples: 54+56 = 110
1b
Mean processed reads
(x10^6)
aligned
not aligned
1c
PC2 (17.35%)
PC1 (19.55%)
Status
Treatment
Control
1d
Treated/Untreated
Ear type
Timepoint
Standardised expr. value (rpmmm, log10)
-4.4
-1.76
0.88
3.51
6.15
1e
Fold change (log2)
Expression value (rpmmm, log10)
Status
Treatment
Control
1f
Residual 67.2%
Individual 8.2%
Timepoint:Individual 7.6%
Treated/Untreated:Individual 7.6%
Treated/Untreated 4.7%
Treated/Untreated:Left ear/Right ear 2%
Left ear/Right ear 1.1%
Left ear/Right ear:Timepoint 0.8%
Timepoint 0.4%
Treated/Untreated:Timepoint 0.3%
Observed variance (%)

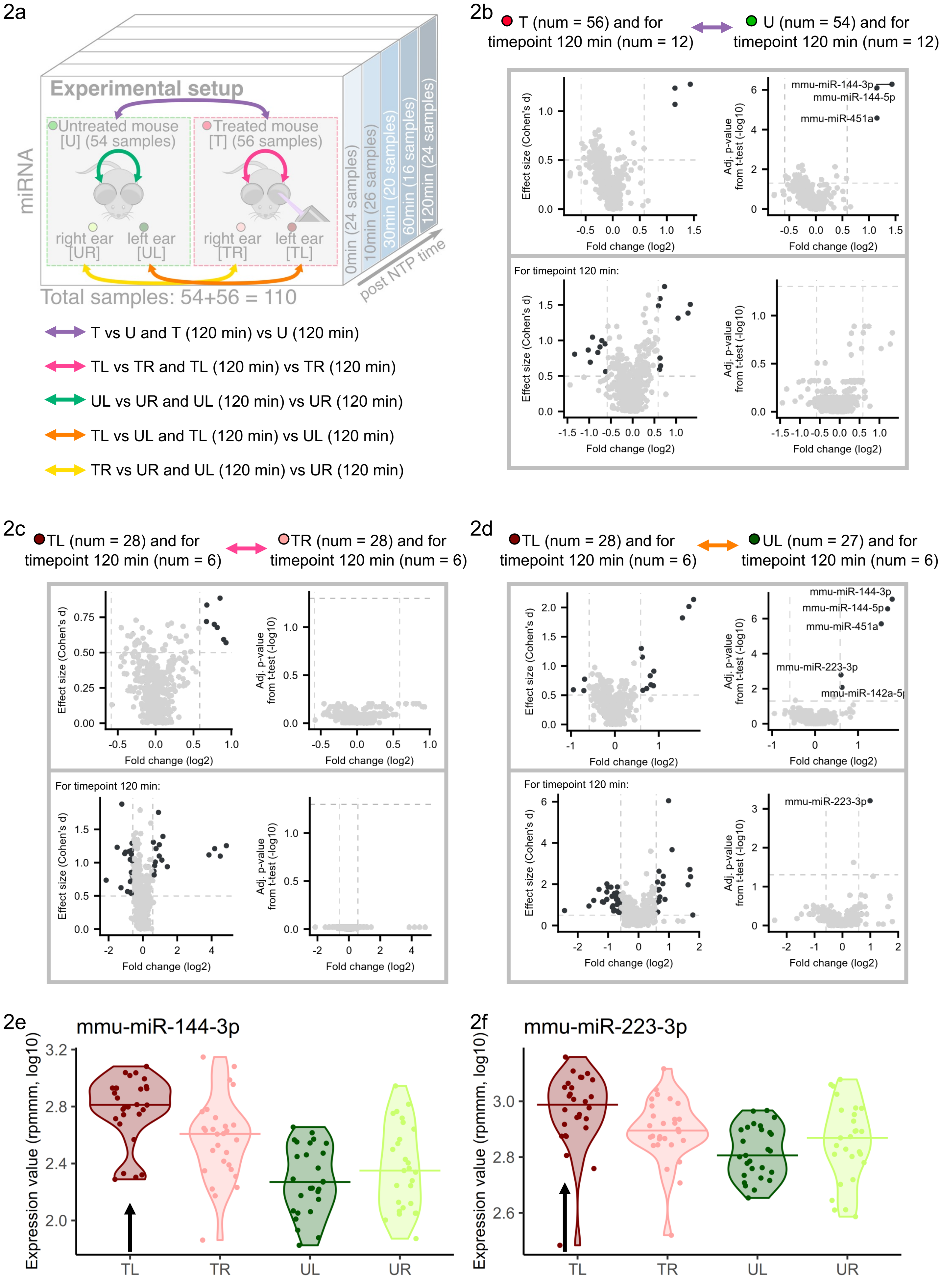
2a
Experimental setup
Untreated mouse
[U] (54 samples)
Treated mouse
[T] (56 samples)
right ear
[UR]
left ear
[UL]
right ear
[TR]
left ear
[TL]
miRNA
0min (24 samples)
10min (26 samples)
30min (20 samples)
60min (16 samples)
120min (24 samples)
post NTP time
Total samples: 54+56 = 110
T vs U and T (120 min) vs U (120 min)
TL vs TR and TL (120 min) vs TR (120 min)
UL vs UR and UL (120 min) vs UR (120 min)
TL vs UL and TL (120 min) vs UL (120 min)
TR vs UR and UL (120 min) vs UR (120 min)
2b
T (num = 56) and for timepoint 120 min (num = 12)
U (num = 54) and for timepoint 120 min (num = 12)
Effect size (Cohen's d)
Adj. p-value from t-test (-log10)
Fold change (log2)
mmu-miR-144-3p
mmu-miR-144-5p
mmu-miR-451a
For timepoint 120 min:
2c
TL (num = 28) and for timepoint 120 min (num = 6)
TR (num = 28) and for timepoint 120 min (num = 6)
Effect size (Cohen's d)
Adj. p-value from t-test (-log10)
Fold change (log2)
For timepoint 120 min:
2d
TL (num = 28) and for timepoint 120 min (num = 6)
UL (num = 27) and for timepoint 120 min (num = 6)
Effect size (Cohen's d)
Adj. p-value from t-test (-log10)
Fold change (log2)
mmu-miR-144-3p
mmu-miR-144-5p
mmu-miR-451a
mmu-miR-223-3p
mmu-miR-142a-5p
For timepoint 120 min:
mmu-miR-223-3p
2e
mmu-miR-144-3p
Expression value (rpmmm, log10)
TL
TR
UL
UR
2f
mmu-miR-223-3p
Expression value (rpmmm, log10)
TL
TR
UL
UR

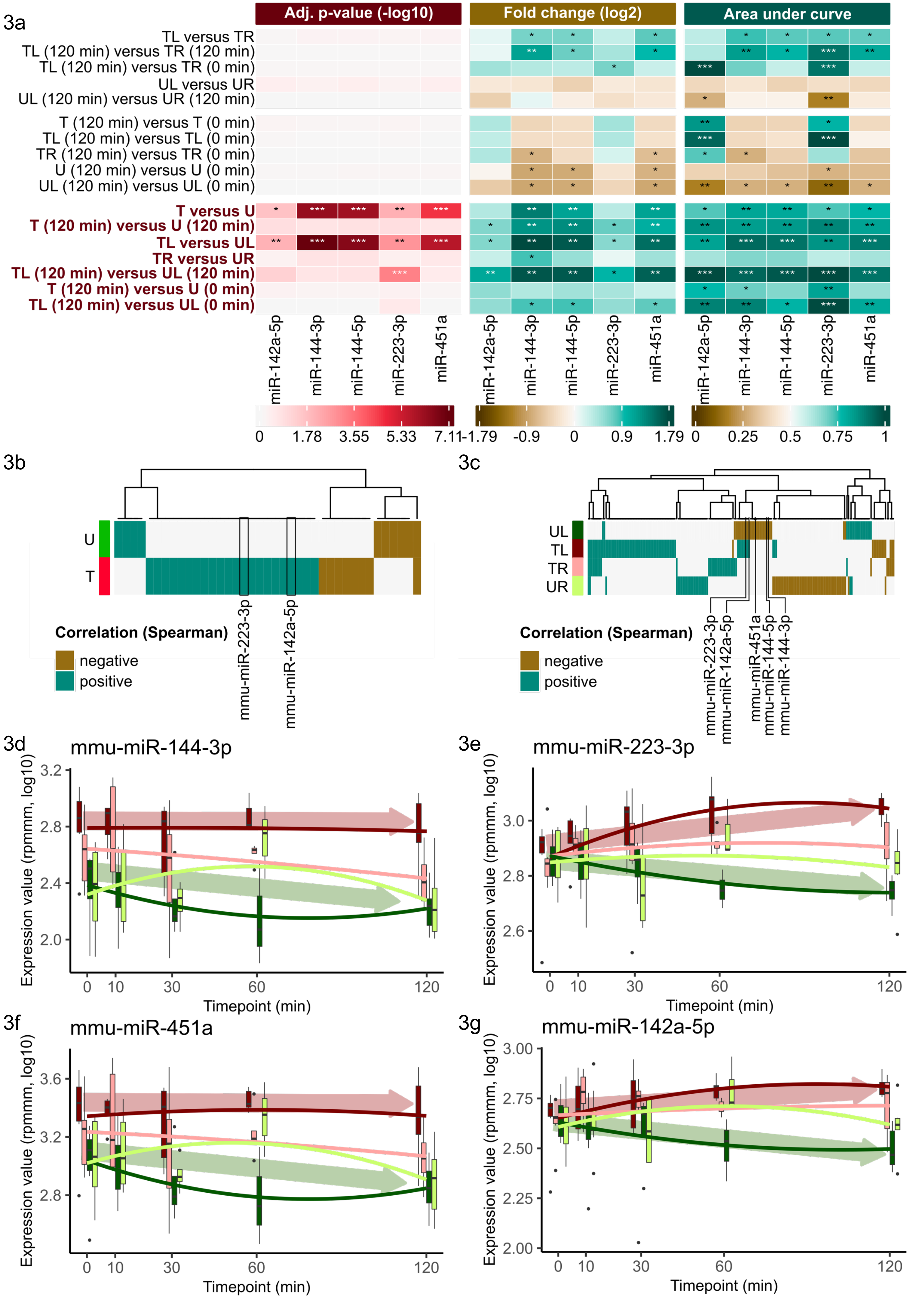
3a
Adj. p-value (-log10)
Fold change (log2)
Area under curve
TL versus TR
TL (120 min) versus TR (120 min)
TL (120 min) versus TR (0 min)
UL versus UR
UL (120 min) versus UR (120 min)
T (120 min) versus T (0 min)
TL (120 min) versus TL (0 min)
TR (120 min) versus TR (0 min)
U (120 min) versus U (0 min)
UL (120 min) versus UL (0 min)
T versus U
T (120 min) versus U (120 min)
TL versus UL
TR versus UR
TL (120 min) versus UL (120 min)
T (120 min) versus U (0 min)
TL (120 min) versus UL (0 min)
miR-142a-5p
miR-144-3p
miR-144-5p
miR-223-3p
miR-451a
0 1.78 3.55 5.33 7.11
-1.79 -0.9 0 0.9 1.79
0 0.25 0.5 0.75 1
3b
U
T
mmu-miR-223-3p
mmu-miR-142a-5p
Correlation (Spearman)
negative
positive
3c
UL
TL
TR
UR
mmu-miR-223-3p
mmu-miR-142a-5p
mmu-miR-451a
mmu-miR-144-5p
mmu-miR-144-3p
Correlation (Spearman)
negative
positive
3d
mmu-miR-144-3p
Expression value (rpmmm, log10)
3.2 2.8 2.4 2.0
0 10 30 60 120
Timepoint (min)
3e
mmu-miR-223-3p
Expression value (rpmmm, log10)
3.0 2.8 2.6
0 10 30 60 120
Timepoint (min)
3f
mmu-miR-451a
Expression value (rpmmm, log10)
3.6 3.2 2.8
0 10 30 60 120
Timepoint (min)
3g
mmu-miR-142a-5p
Expression value (rpmmm, log10)
3.00 2.75 2.50 2.25 2.00
0 10 30 60 120
Timepoint (min)

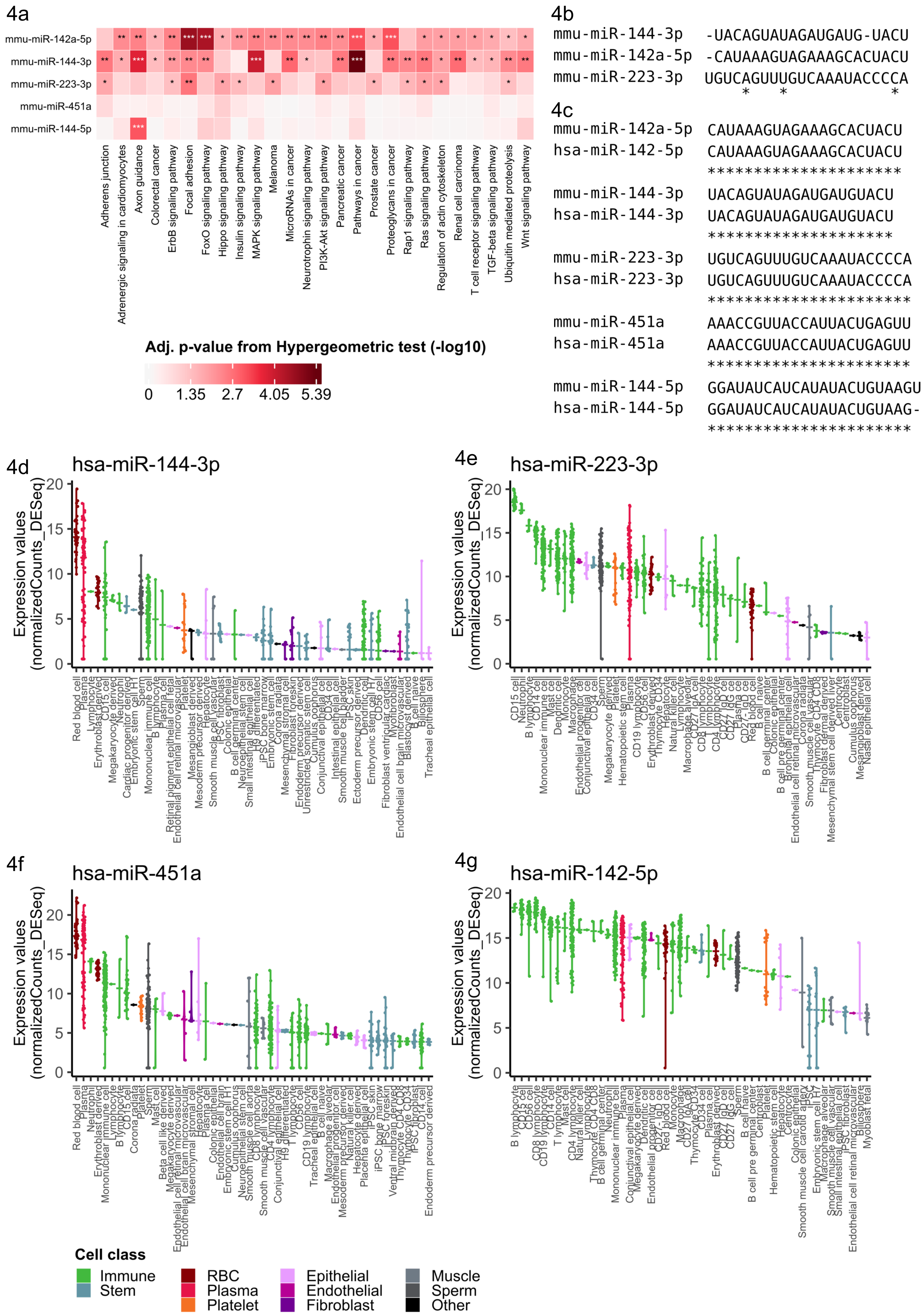
4a
mmu-miR-142a-5p
mmu-miR-144-3p
mmu-miR-223-3p
mmu-miR-451a
mmu-miR-144-5p
Adherens junction
Adrenergic signaling in cardiomyocytes
Axon guidance
Colorectal cancer
ErbB signaling pathway
Focal adhesion
FoxO signaling pathway
Hippo signaling pathway
Insulin signaling pathway
MAPK signaling pathway
Melanoma
MicroRNAs in cancer
Neurotrophin signaling pathway
PI3K-Akt signaling pathway
Pancreatic cancer
Pathways in cancer
Prostate cancer
Proteoglycans in cancer
Rap1 signaling pathway
Ras signaling pathway
Regulation of actin cytoskeleton
Renal cell carcinoma
T cell receptor signaling pathway
TGF-beta signaling pathway
Ubiquitin mediated proteolysis
Wnt signaling pathway
Adj. p-value from Hypergeometric test (-log10)
0 1.35 2.7 4.05 5.39
4b
mmu-miR-144-3p -UACAGUAUAGAUGAUG-UACU
mmu-miR-142a-5p -CAUAAAGUAGAAAGCACUACU
mmu-miR-223-3p UGUCAGUUUGUCAAAUACCCCA
4c
mmu-miR-142a-5p CAUAAAGUAGAAAGCACUACU
hsa-miR-142-5p CAUAAAGUAGAAAGCACUACU
mmu-miR-144-3p UACAGUAUAGAUGAUGUACU
hsa-miR-144-3p UACAGUAUAGAUGAUGUACU
mmu-miR-223-3p UGUCAGUUUGUCAAAUACCCCA
hsa-miR-223-3p UGUCAGUUUGUCAAAUACCCCA
mmu-miR-451a AAACCGUUACCAUUACUGAGUU
hsa-miR-451a AAACCGUUACCAUUACUGAGUU
mmu-miR-144-5p GGAUAUCAUCAUAUACUGUAAGU
hsa-miR-144-5p GGAUAUCAUCAUAUACUGUAAG-
4d hsa-miR-144-3p
4e hsa-miR-223-3p
4f hsa-miR-451a
4g hsa-miR-142-5p
Expression values (normalizedCounts_DESeq)
Cell class
Immune
Stem
RBC
Plasma
Platelet
Epithelial
Endothelial
Fibroblast
Muscle
Sperm
Other

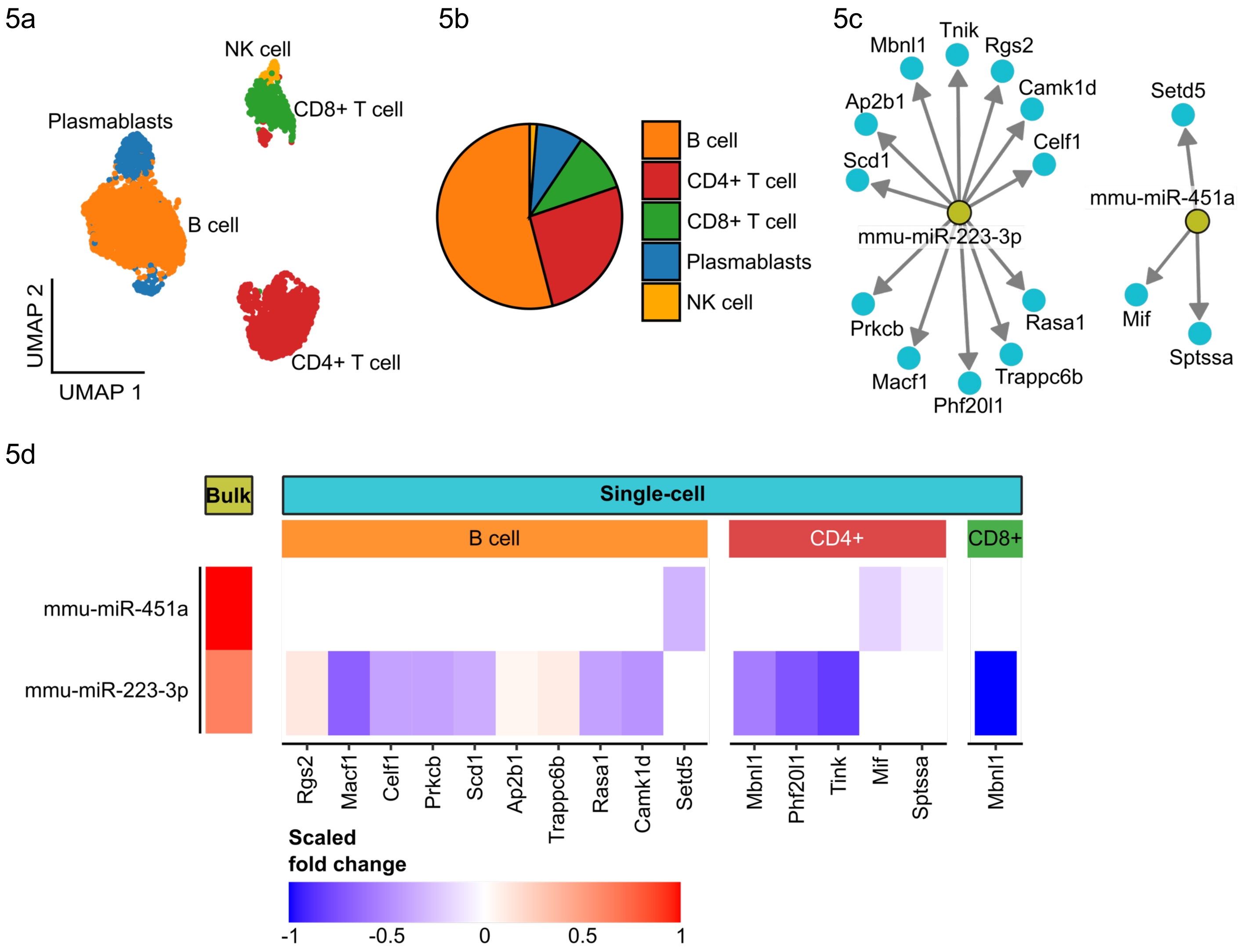

5a
NK cell
CD8+ T cell
Plasmablasts
B cell
CD4+ T cell
UMAP 2
UMAP 1
5b
B cell
CD4+ T cell
CD8+ T cell
Plasmablasts
NK cell
5c
Mbnl1
Tnik
Rgs2
Camk1d
Ap2b1
Celf1
Scd1
mmu-miR-223-3p
Prkcb
Macf1
Phf20l1
Trappc6b
Rasa1
Setd5
mmu-miR-451a
Mif
Sptssa
5d
Bulk
Single-cell
B cell
CD4+
CD8+
mmu-miR-451a
mmu-miR-223-3p
Rgs2
Macf1
Celf1
Prkcb
Scd1
Ap2b1
Trappc6b
Rasa1
Camk1d
Setd5
Mbnl1
Phf20l1
Tink
Mif
Sptssa
Mbnl1
Scaled fold change
-1
-0.5
0
0.5
1
