# Supplementary material for: Skin treatment with non-thermal plasma modulates the immune system through miR-223-3p and its target genes
Source: RNA Biol. 2024 Jun 3;21(1):31–44. doi: 10.1080/15476286.2024.2361571 (PMC11152102; doi:10.1080/15476286.2024.2361571)
Supplement: Supplemental Material [file KRNB_A_2361571_SM2072.zip › Supplemental information.docx]

**Supplemental information**

**Extended Data Fig. 1: a** The percentage of reads, which are mapped to miRNAs. The bottom annotation indicates the sample type and timepoint.

**Extended Data Fig. 2: a** TR versus UR mice comparison. The effect size plots are located on the left side and Volcano plots on the right side of the different boxes with the comparisons from Figure 2a. The upper plots include all samples over all timepoints and the comparison for only the timepoint 120 minutes is depicted in the lower part of the box. Deregulated miRNAs with effect size >= 0.5 or p-value < 0.05 (t-test, -log10) are highlighted in dark grey**. b** UL versus UR mice comparison analogous to Supplementary Figure 2a.

**Extended Data Fig. 3: a** Adjusted (layout changes and display only four cell types) plot from the web tool of Juzenas *et al.*^40^. This data set is offering a comprehensive, cell specific miRNA catalogue of selected PBMC types.
